# Pediatric ACL Injuries: A Review of Current Concepts

**DOI:** 10.2174/1874325001711010378

**Published:** 2017-04-28

**Authors:** Vikas Trivedi, Panna Mishra, Deepankar Verma

**Affiliations:** 1 Department of Orthopedics, Era’s Lucknow Medical College, Lucknow, India; 2 Hind Institute of Medical Sciences, Lucknow, India; 3 Department of Orthopedics, Subharti Medical College, Meerut, India

**Keywords:** Adolescent, ACL injury, ACL reconstruction, Treatment algorithm, Post op rehabilitation, Injury prevention

## Abstract

**Background:**

The number of anterior cruciate ligament (ACL) injuries reported in skeletally immature athletes has increased over the past 2 decades. The reasons for this increased rate include the growing number of children and adolescents participating in competitive sports vigorous sports training at an earlier age and greater rate of diagnosis because of increased awareness and greater use of advanced medical imaging. There is a growing need for a consensus and evidence based approach for management of these injuries to frame a dedicated age specific treatment strategy.

**Methods:**

This article does a systematic evidence based literature review of management of Pediatric ACL injuries seen in several forms: tibial eminence avulsion fractures partial ACL tears and full thickness ligament tears and its outcome analysis.

**Results:**

The mechanism of Safe and effective surgical techniques for children and adolescents with ACL injuries continues to evolve. The numerous age matched techniques are extensively discussed. Neuromuscular training can reduce the risk of ACL injury in adolescent girls.

**Conclusion:**

This review outlines the current state of knowledge on diagnosis treatment and prevention of ACL injuries in children and adolescents and helps in guiding the treatment through a dedicated algorithm.

## INTRODUCTION

Pediatric and adolescent Anterior cruciate ligament (ACL) injuries are becoming increasingly common throughout the world as more and more children are engaging in physical activity and competitive sports. Recent Literature point to a high incidence of ACL injuries in skeletally immature athletes and show that these injuries account for about 31% of all knee injuries in soccer players between the ages of 5 and 18 years [1]. Another study [2] reports an incidence of ACL tear of 47% in preadolescents and 65% of adolescents who present with an acute knee hemarthrosis. There are 4 types of ACL tears commonly seen in pediatric population [1] Cartilagenous avulsions [2]. Bony avulsion or tibial eminence fracture [3] Mid substance complete tears and [4] Partial tears, this varied spectrum of injuries can make accurate diagnosis difficult. The other important challenges in this age group are potential risk of recurrent instability, secondary chondral and meniscal injuries following non operative treatment [3, 4] and the risks associated with surgical treatment due to vulnerability of open epiphyseal growth plates [5-8]. Further, if surgical treatment is chosen there is controversy over the appropriate method of reconstruction as well as selection of the graft material and its fixation. Finally, the post-operative rehabilitation protocol can be tricky in the pediatric patients with respect to proper adherence with increased risk of re-ruptures.

The aim of this review paper is to [1] discuss the patho-biomechanics of ACL injury in pediatric patients [2] outline the preoperative diagnostic evaluation of a pediatric patient with a suspected ACL injury [3] highlight the unique anatomical and physiological characteristics related to ACL reconstruction [4] outline the current evidence based management of these injuries [5] Discuss specific post-operative rehabilitation protocols and finally to review the age specific injury prevention treatment strategies.

## BIOMECHANICS OF PEDIATRIC ACL INJURY

Most commonly, ACL injuries are non-contact injuries which are caused due to a pivot mechanism with the knee partially flexed and the foot planted on the ground [9], sometimes hyperextension of the knee with a valgus or rotational force has been noted and another study pointed that tibial eminence fractures are more likely to occur than ACL tears when the knee loading rates were slower [10, 11]. Also, it has been noted with these fractures there is variable degree of plastic deformation with permanent elongation of ACL fibers which may cause residual clinical laxity despite anatomic reduction and healing of fracture [10-12]

## EVALUATION OF THE PEDIATRIC ACL INJURY

The ACL injured children usually give a history of a non-contact injury with an audible pop followed by a rapidly developing hemarthrosis and difficulty in returning to sport initially but with time these young athletes ignore the injury and continue playing sports with recurrent episodes of instability and often return to physician only when experience extreme pain or severe locking due to secondary chondral or meniscal injuries [3, 4]. The weight bearing period after ACL injury is typically delayed in children as compared to adults.

**Fig. (1):**
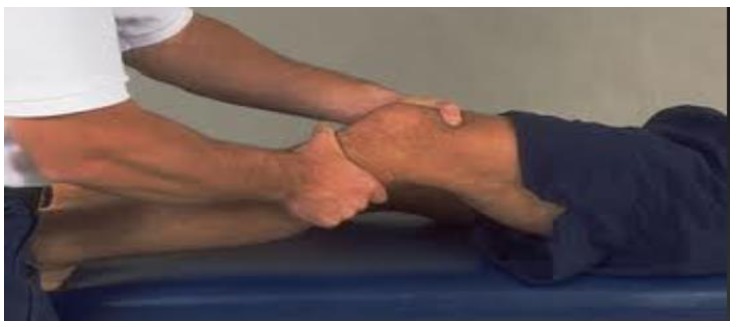
The Lachman Test.

In children presenting with acute traumatic hemarthrosis and severe pain it is better to take radiographs first before the clinical exam to rule out a tibial eminence fracture. The clinical examination of these patients is essentially on the same lines as the adults though it is more difficult to perform. A complete and careful Knee examination in comparison to the opposite knee is a very important tool in establishing the correct diagnosis of the ACL tear. In a relaxed patient Lachman (Fig. **1**) and pivot shifts test are reliable indicators of ACL injury, though some studies recommend pivot shift test to be better predictor for injury and also an important aid in deciding the surgical treatment [13]. Through range of motion assessment, joint line palpation, McMurrays testing, careful physeal palpation, varus, valgus stress tests, patellar stability tests are done to rule out other associated meniscal, chondral, physeal, patellar and ligamentous injuries which have an important bearing in the choice of the treatment to be employed.

**Fig. (2):**
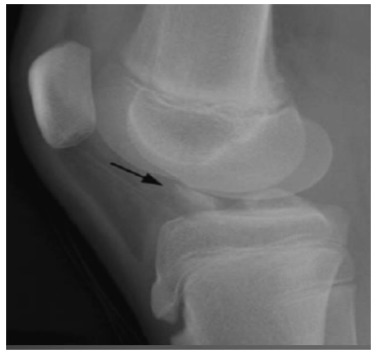
The perfect lateral X-ray to diagnose tibial eminence fracture.

**Fig. (3):**
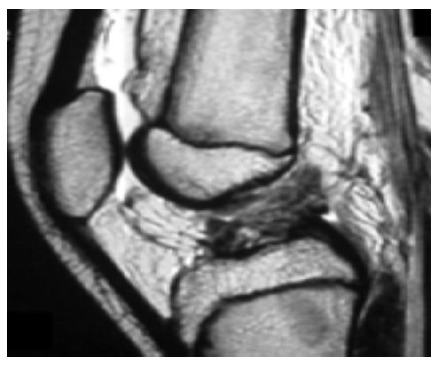
Sagittal MRI showing a complete ACL tear in a pediatric patient.

Knee series X-rays are done which include the AP, Lateral, notch/tunnel and skyline views. In children with less pain, AP and Lateral radiographs can be performed in weight bearing position and special emphasis is given to obtain a perfect lateral X ray to rule out a tibial eminence fracture (Fig. **2**). MR Imaging is a very useful tool in diagnosing the ACL tear in an acute setting when the clinical exam is difficult and has shown to increase the sensitivity and specificity of diagnosing a complete ACL tear to above 90% [14, 15]. (Fig. **3**) MRI is extremely useful in picking up associated meniscal, chondral and ligamentous injuries, cartilagenous avulsion of ACL, Physeal separations and an undisplaced Type 1 tibial eminence fracture. The sensitivity of MRI in detecting partial ACL tears is poor [16, 17]. Diagnosing a partial tear is difficult and requires a combined careful pivot shift test evaluation and detailed MRI analysis to formulate a correct treatment plan.

## ASSESSMENT OF SKELETAL MATURITY

**Fig. 4:**
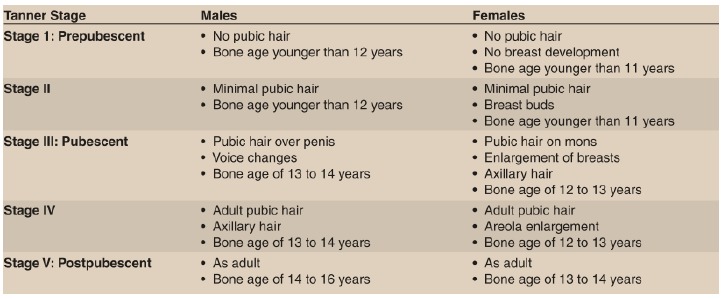
Tanner staging chart.

A careful maturity assessment with respect to remaining growth potential is extremely important to plan the correct treatment of ACL injuries; the main dilemma is whether surgical treatment should be provided before patients reach skeletal maturity or whether non-operative treatment should be provided until the physis has closed. Surgical reconstruction risks physeal damage and resultant angular deformity and limb shortening, while delaying surgery may increase menisci and cartilage damage. The best physiological age assessment method is Tanner staging [18] (Fig. **4**) and to estimate the skeletal age, left Hand PA X ray is taken and compared by Greulich and Pyle atlas [19, 20]. Both methods give reliable indication of remaining growth left and help in planning the correct treatment choice. MRI also has been suggested to be a good radiation-free method to determine skeletal maturity in the future, although further validation of this measure as well as cost benefit analysis is needed [21, 22].

## MANAGEMENT STRATEGIES

The treatment of ACL tears in the pediatric patients remains challenging and controversial. An ACL tear in a child is not a true orthopedic emergency. In the past, many orthopedic surgeons preferred to delay surgical treatment till skeletal maturity; treatment consisted of bracing, rehabilitation, and sports restriction for many months with satisfactory short-term results [23-25]. For most pediatric athletes, conservative management may still be a reasonable treatment option.

However, many pediatric athletes and their parents are less inclined to agree to restrict the athlete’s activity. In such cases, an ACL tear in the pediatric athlete treated conservatively can lead to additional instability episodes, meniscal tears, articular cartilage damage, and early-onset arthritis [4, 25-28]. Therefore, most recent literature now supports early surgery for pediatric athletes with an ACL- deficient knee and recurrent episodes of instability [27, 29-31]. As per current evidence, ACL surgery is about 90% successful in restoring knee stability and patient satisfaction [32].

## NON OPERATIVE TREATMENT

As per current evidence, non-operative treatment is offered to pediatric patients with partial tears with appreciable growth left, those with a negative pivot shift test and with less than 50% of fibers ruptured [5, 9, 33]. Non operative treatment consists of a dedicated knee physical therapy program as well as proprioception/neuromuscular re-education program and careful bracing. This involves close clinical supervision with a view for any recurrent instability or precipitation of meniscal/chondral injuries.

Type 1, non-displaced tibial eminence fractures (McKeever Classification) are treated by immobilization in a above knee cast in 10-20 degrees of knee flexion [34-36]. Type 2 tibial eminence fractures with small displacement, reduction can be achieved with aspiration of hemarthrosis in some cases and then cast can be applied.

## SURGICAL TREATMENT

Multiple timely discussions with the parents and the child about the appropriate surgical management options, careful assessment of skeletal maturity and associated intra-articular injuries assessment and understanding their goals and expectations are very important.

The general indications for surgery are (1): the patient’s inability to participate in his or her chosen sport (2) instability that affects activities of daily living, (3) an associated treatable meniscal tear or chondral lesion and (4) a knee injury with multiple torn ligaments.

Most orthopedic surgeons agree that the optimal surgical timing of ACL reconstruction is after full range of knee motion has been achieved, unless in the setting of a Tibia eminence fracture, bucket handle meniscal teal and multiple ligamentous injury [37, 38].

## SURGICAL TECHNIQUE

There are multiple techniques described in the literature ranging from extra articular reconstructions to all epiphyseal to transphyseal reconstructions with various hybrid techniques and graft choices. To simplify, these techniques can be divided into physeal sparing and transphyseal reconstructions.

The decision to perform the type of reconstruction and the graft choice should be determined by the remaining growth potential left as determined by the Tanner staging [18] and determination of skeletal age by hand radiographs [19, 20]. The careful usage of both these techniques in combination to plan the type of reconstruction is recommended.

## TUNNEL & GRAFT CONSIDERATIONS

The unique and important considerations in pediatric patients are the effect of tunnel size and the tunnel drill angle on the physis. The risk of physeal arrest increases when the physeal damage area affected by drilling increases more than 7% [39] and also the risk of arrest increases when the tunnel drill angle becomes more oblique [40]. Graft tension is also an important determinant of physeal damage, the higher the tension, the more is the risk of physeal injury [41]. Allograft usage is also shown to increase re-rupture rates [42].

Literature suggests that small diameter tunnels with soft tissue autografts, vertical tunnel placement, avoiding implant or hardware fixation across the lateral distal femoral physis and less graft tensioning minimizes growth disturbances [39-42].

The ideal technique would be taking into these considerations as well as employing suitable mechanical modifications to ensure a stable, strong and physiological ACL reconstruction to ensure a rotationally and translationally stable knee.

## PLANNING

The treatment algorithms which aid in planning are described by Milewski *et al*. [43] is based on the skeletal age and recommends Mitchelli-Kocher technique [44] of combined intra and extra articular physeal sparing reconstruction for a skeletal age of 6, the modified Anderson technique for skeletal age of 8 years, the Ganley- Lawrence all Epiphyseal technique for skeletal age of 10 [45], a hybrid technique for skeletal age of 12 and a transphyseal technique similar to adults for patients with skeletal age of 14 years or more.

Another very useful recent algorithm proposed by Fabricant *et al.* [46] takes into account various patient derived variables and skeletal age to appropriately determine the treatment course.

The treatment algorithm which can be employed is displayed in Fig. (**5**).

**Fig. (6):**
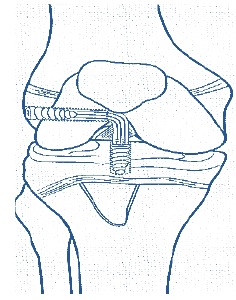
Diagram of Ganley-Lawrence all epiphyseal all inside technique.

**Fig. (7):**
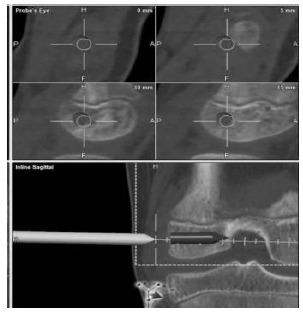
Navigated physeal tunnel placement.

We prefer the Ganley Lawrence technique [45] of Arthroscopic. All epiphyseal inside hamstring autograft technique in prepubescent children (Fig. **6**) with recent usage of O- arm and Navigation systems (Fig. **7**) these all-epiphyseal all-inside techniques, are becoming more precise and easier to perform.

## POST ACL RECONSTRUCTION REHABILITATION

Rehabilitation after ACL reconstruction needs to be individualized for each patient’s functional and sports ability demands and also tailored to the particular surgical procedure used. In general, a graduated rehabilitation program (usually a 5 Phase program) emphasizing full extension; immediate weight bearing; active range of motion; and strengthening of the quadriceps, hamstrings, hip, and core can be started in the first few weeks after surgery. Progressive re-habilitation during the first 3 months after surgery includes range-of-motion exercises, patellar mobilization, proprioceptive exercises, endurance training, and closed-chain strengthening exercises. Straight-line jogging, plyometric exercises, and sport-specific exercises are added after 4 to 6 months. Return to Sports can be achieved by 6-9 months.

## DISPLACED IRREDUCIBLE TIBIAL EMINENCE FRACTURE

Numerous operative techniques are described for reduction and fixation of displaced tibial eminence fractures including open [47] or arthroscopic [35, 36] which employ different fixation implants like sutures, metal screws, bioabsorbable nails, K wires and suture anchors.

**Fig. (8):**
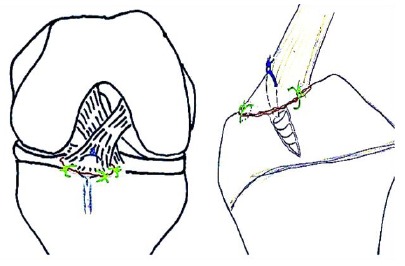
Hybrid All inside fixation of displaced Tibial eminence fracture by a suture anchor (Type III A or Type III B).

We prefer the arthroscopic hybrid all inside repair technique (Physeal sparing) using suture anchors recently described by Kim *et al.* [48], this is potentially safe for physes and affords rigid stable fixation (Fig. **8**).

## POST FIXATION REHABILITATION

Long leg splint was applied for 3 days, and the range of motion of the knee was gradually increased, followed by partial weight bearing for the next 4 weeks. Full weight bearing as tolerated after radiological union and return to sports after adequate strengthening usually after 3 months.

## PREVENTION OF ACL INJURIES

The development of the pediatric athlete’s neuromuscular control, has been shown to be a modifiable risk factor for developing an ACL tear. There are two recognized prevention programs, the FIFA-11 [49, 50] and the PEP (Prevent injury and Enhancement Program) from Santa Monica [51], which are designed to improve the athlete’s neuromuscular control and, in turn, prevent ACL injuries. For young girls, the best window of opportunity for ACL injury risk reduction may be during early pubertal maturation, at or just before girls’ neuromuscular risk factors start to become evident and ACL injury rates in girls dramatically increase. In the clinical setting, we can use a simple drop vertical jump test to help us identify athletes at higher risk for ACL injury based on their landing mechanics and then recommend these individuals to designated therapists and training centers to work on their neuromuscular control.

## CONCLUSION

1. Anterior cruciate ligament injuries in pediatric patients are increasingly common. They can present as tibial eminence fractures, partial ACL injuries, and complete ACL tears.
2. MRI can be valuable for diagnosing ACL tears and associated meniscal and chondral injury in the pediatric athlete whose physical examination is difficult to perform because of pain, swelling and lack of cooperation.
3. An ACL tear in a pediatric patient is not a surgical emergency. Multiple discussions with the patient and parents are done with respect to the athlete’s goals and parental expectations and to educate the family about possible treatment options.
4. The patient’ s skeletal age, measured by an anteroposterior radiograph of the left hand and wrist, and Tanner stage are important tools in planning the ideal surgical treatment technique.
5. Surgical treatment has led to improved results in those with displaced eminence fractures, partial tears with a positive pivot shift under anesthesia, and complete ACL tears.
6. Technique for ACL reconstruction is typically based on the status of the physis and various treatment algorithm guides are available to decide the best surgical option.
7. Neuromuscular training appears to reduce the risk of injury in adolescent female and male athletes. Prevention training that incorporates plyometric and strengthening exercises, combined with feedback to athletes on proper technique, appears to be most the effective.

## Figures and Tables

**Fig. (5):**
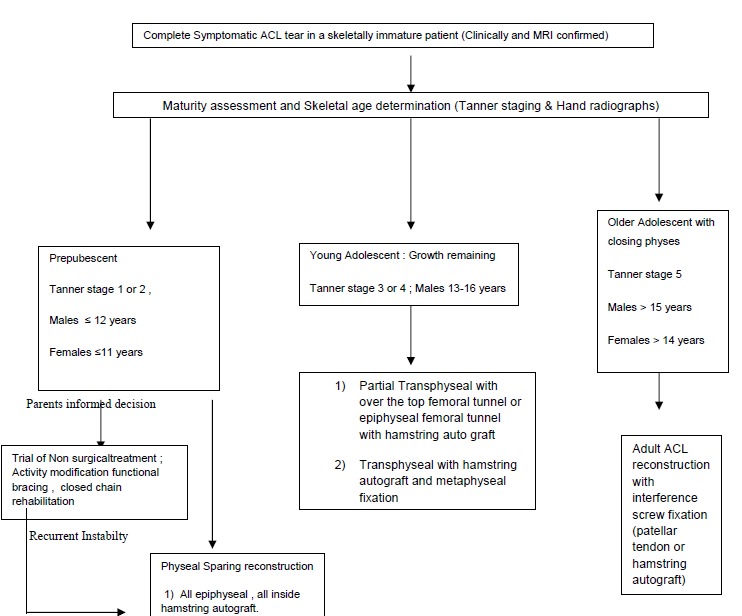
Treatment algorithm for guiding ACL reconstruction in a pediatric athlete.

## LIST OF ABBREVIATIONS

ACL: = Anterior cruciate ligament
FIFA: = Fédération internationale de football association
K wire: = Kirschner wire
MRI: = Magnetic resonance imaging
PEP: = Prevent injury and enhancement program
